# Interferon gamma effect on immune mediator production in human nerve cells infected by two strains of *Toxoplasma gondii*

**DOI:** 10.1051/parasite/2015039

**Published:** 2015-12-21

**Authors:** Nour Mammari, Philippe Vignoles, Mohamad Adnan Halabi, Marie-Laure Dardé, Bertrand Courtioux

**Affiliations:** 1 Univ. Limoges, UMR-S 1094, Tropical Neuroepidemiology, Institute of Neuroepidemiology and Tropical Neurology, CNRS FR 3503 GEIST 87000 Limoges France; 2 UMR CNRS 7276, FR 3503 GEIST, Faculty of Pharmacy, University of Limoges 87000 Limoges France; 3 CHU Limoges, Department of Parasitology, and Biological Resource Centre for Toxoplasma 87000 Limoges France

**Keywords:** *Toxoplasma gondii*, Human nerve cells, IFN-γ, Immunity

## Abstract

Interferon gamma (IFN-γ) is the major immune mediator that prevents toxoplasmic encephalitis in murine models. The lack of IFN-γ secretion causes reactivation of latent *T. gondii* infection that may confer a risk for severe toxoplasmic encephalitis. We analyse the effect of IFN-γ on immune mediator production and parasite multiplication in human nerve cells infected by tachyzoites of two *T. gondii* strains (RH and PRU). IFN-γ decreased the synthesis of MCP-1, G-CSF, GM-CSF and Serpin E1 in all cell types. It decreased IL-6, migration inhibitory factor (MIF) and GROα synthesis only in endothelial cells, while it increased sICAM and Serpin E1 synthesis only in neurons. The PRU strain burden increased in all nerve cells and in contrast, RH strain replication was controlled in IFN-γ-stimulated microglial and endothelial cells but not in IFN-γ-stimulated neurons. The proliferation of the PRU strain in all stimulated cells could be a specific effect of this strain on the host cell.

## Abstract (fr)

L’interféron gamma (IFN-γ) est le médiateur immunitaire majeur qui empêche le développement de l’encéphalite toxoplasmique dans le modèle murin. L’absence de sécrétion d’IFN-γ provoque la réactivation de l’infection latente à *T. gondii*, avec un risque de développement d’une toxoplasmose cérébrale sévère. Nous avons analysé l’effet de l’IFN-γ sur la production des médiateurs immunitaires et la multiplication parasitaire dans des cellules nerveuses humaines infectées par des tachyzoïtes de deux souches de *T. gondii* (RH et PRU). L’IFN-γ diminue la synthèse de MCP-1, G-CSF, GM-CSF et Serpin E1 dans tous les types cellulaires. Il diminue la synthèse d’IL-6, de MIF et de GROα uniquement dans les cellules endothéliales, tandis qu’il stimule la synthèse de sICAM et de Serpin E1 dans les neurones. La charge parasitaire de la souche PRU augmente dans toutes les cellules nerveuses. En revanche, la multiplication de la souche RH est contrôlée dans les cellules microgliales et endothéliales stimulées par l’IFN-γ, mais pas dans les neurones stimulés par l’IFN-γ. La prolifération de la souche PRU dans toutes les cellules stimulées pourrait être un effet spécifique de cette souche sur la cellule hôte.

## Introduction

*Toxoplasma gondii* induces a potent cellular immune response that is essential for controlling infection. Interferon gamma (IFN-γ) is one of the most important cytokines for immune control of *Toxoplasma* infection in mice. In infected mice, this cytokine could control tachyzoite proliferation, thus maintaining latency of chronic infection in the brain and preventing toxoplasmic encephalitis [26]. During acute *Toxoplasma* infection, natural killer (NK) cells, CD4^+^ and CD8^+^ T cells are the major sources of IFN-γ and this cytokine might stimulate all effector cells to activate a protective immune response against *T. gondii* infection [7]. In the murine central nervous system (CNS), IFN-γ activates neuronal cells to control tachyzoite multiplication [25]. The stimulation of murine microglial cells with IFN-γ and TNFα inhibited the penetration of PLK tachyzoites (mouse non-virulent type II *Toxoplasma* strain) [14]. Intracellular type I and II tachyzoite proliferation is inhibited by pre-treatment with IFN-γ of both human and murine astrocyte cells [15, 20]. In addition, it was demonstrated that IFN-γ is involved in *Toxoplasma* cyst formation in murine astrocytes and neurons [12, 18].

Most of these data were obtained in murine models. In a previous paper, we reported the production of cytokines and chemokines by infected human nerve cells in the absence of IFN-γ [19]. To further study the role of IFN-γ in the control of *Toxoplasma* infection in human nerve cells *in vitro*, we report the effect of IFN-γ on the activation of infected human nerve cells and the control of intracellular multiplication of two *T. gondii* strains.

## Materials and methods

### Parasite production

One type I strain (RH) and one type II strain (PRU) of *T. gondii* were used in the present study. Tachyzoites of each strain were grown and purified in human fibroblastic cell cultures (MRC5) as previously described [19]. All protocols involving animals were approved by the Committee on the Ethics of Animal Experiments of Limousin, France (Permit No. 3-07-2012).

### Human nerve cell cultures

Human microglial cells (CMH5) (kindly provided by Pr. P. Vincendeau, Bordeaux, France) [16], human bone marrow endothelial cells (Hbmec) (obtained from the cell line established by Pr. D. Paulin, University of Paris 7, France) [24] and human neuroblastoma cells (SH SY5Y) (kindly provided by Pr. M.O. Jauberteau-Marchan, Limoges, France) [21] were cultured following the protocol described in the previous study [19].

### Treatment and infection of human nerve cells

Each type of cell culture was treated with IFN-γ (100 ng/mL) (Sigma-Aldrich) for 24 h. A total of 10^6^ human nerve cells (confluent cells) were then infected separately for 24 h by tachyzoites either from the RH strain (type I) or from the PRU strain (type II) with a ratio of one cell for two tachyzoites. Control cells were uninfected and non-stimulated cells, and uninfected stimulated cells. Each experiment was performed in triplicate at separate times to ensure reproducibility of results.

### Cytokine, chemokine and growth factor analysis

The pro-inflammatory proteins were analysed in co-culture supernatants using the Proteome Profiler Array technique (R&D, Lille, France), following the protocol described in the previous study [19].

### Quantification of parasite burden

The viable parasite burden was quantified in the pellet cell culture by semi-quantitative reverse transcription polymerase chain reaction (RT-PCR) using specific primers for *T. gondii* 529 gene repeat. qRT-PCR was performed using a One Step SYBR Green RT-PCR Kit (Qiagen, Paris, France). Each RNA sample (≤ 100 ng) was added to PCR tubes containing SYBR master mix (12.5 μL), RT mix (0.25 μL) and specific primers (0.6 μM) [sense: 5′-AGGCGAGGTGAGGATGA-3′; antisense: 5′-TCGTCTCGTCTGGATCGAAT-3′] (Sigma-Aldrich) [4]. Quantification was performed using a dose of tachyzoites ranging from 5 × 10^4^ to 1 × 10^4^ *Toxoplasma* amplified by primers for the 529 pb repeat gene [19].

### Statistical analysis

Statistical analysis of pro-inflammatory protein profiles and comparison of parasite burden quantification were carried out using the Scheirer-Ray-Hare test. All interactions between the different factors were performed using Steel-Dwass and Siegel-Castellan tests with a 95% confidence level.

## Results

Among the 36 immune mediators tested, two interleukins (IL-6 and IL-8), three chemokines (MCP-1, migration inhibitory factor [MIF], GROα) and five other markers (G-CSF, GM-CSF, Serpin E1 and sICAM) were significantly expressed in IFN-γ stimulated and non-stimulated CMH5, Hbmec and SH SY5Y infected cells [19].

Compared to immune mediator expression in infected non-stimulated cells [19], we observed that stimulation by IFN-γ leads to a decrease in IL-6 and GROα levels in RH- and PRU-infected endothelial cells (*p* < 0.05) (Table 1) and MCP-1, G-CSF, GM-CSF and Serpin E1 synthesis in the three cell types (Table 1).

**Table 1.** Cell types, parasite strains and IFN-γ effects on pro-inflammatory protein expressions. Pro-inflammatory proteins were analysed in supernatants of cultivated RH- or PRU-infected cells using Proteome Profiler Array. All spots were quantified as pixel units by GeneTools. Quantification corresponds to the pixel values of uninfected cells (negative control) subtracted from pixel values of infected cells. The effect level was determined using the Scheirer-Ray-Hare test and Steel-Dwass and/or Siegel-Castellan post hoc tests. ↘ expression decrease with *p* < 0.05; ↘↘ expression decrease with *p* < 0.001. ↗ expression increase with *p* < 0.05; ↗↗ expression increase with *p* < 0.001.

Cells		CMH5		Hbmec		SH SY5Y	
	Strains proteins	RH	PRU	RH	PRU	RH	PRU
Interleukins	IL-6	–	–	↘	↘	–	–
	IL-8	–	–	–	–	–	–
Chemokines	MIF	–	–	–	↘	–	–
	MCP-1	–	↘↘	–	↘↘	↘	–
	GROα	–	–	–	↘	–	–
Growth factors	G-CSF	↘↘	↘↘	↘↘	↘↘	↗	↘
	GM-CSF	–	↘↘	↘	–	–	↘
Other	SlCAM	–	↘↘	–	↘↘	↗↗	↗↗
	Serpin El	–	↘↘	–	↘↘	↗↗	↗↗

More specifically, the level of IL-6 synthesis is decreased only in RH- and PRU-infected and stimulated endothelial cells (*p* < 0.05) (Table 1). For chemokines, we noted that MIF is decreased in stimulated and PRU-infected endothelial cells (*p* < 0.05) (Table 1). Monocyte chemoattractant protein 1 (MCP-1) synthesis is decreased in IFN-γ-stimulated and PRU-infected microglial cells (CMH5) (*p <*  0.001) and endothelial cells (Hbmec) (*p* < 0.001). In neurons (SH SY5Y), MCP-1 levels decreased only in RH-infected and stimulated cells (*p* < 0.05) (Table 1). In the presence of IFN-γ, G-CSF synthesis decreased significantly in RH- and PRU-infected microglial (*p* < 0.001) and endothelial cells (*p* < 0.001) and only in PRU-infected neurons (*p* < 0.05). GM-CSF production was significantly decreased in PRU-infected microglial cells (*p* < 0.001) and in RH-infected endothelial cells (*p* < 0.05) and PRU-infected neurons (*p* < 0.05). In the presence of IFN-γ, we also noted that sICAM, adhesion protein decreased synthesis in PRU-infected microglial (*p* < 0.001) and endothelial cells (*p* < 0.001) (Table 1) but increased in RH- and PRU-infected neurons (*p* < 0.001). IFN-γ also had a significant effect on Serpin E1 protein production; it decreased synthesis in PRU-infected microglial (*p* < 0.001) and endothelial cells (*p* < 0.001) and increased in PRU- and RH-infected neurons (*p* < 0.001). In contrast, IFN-γ did not have a significant effect on IL-8.

**Figure 1.**
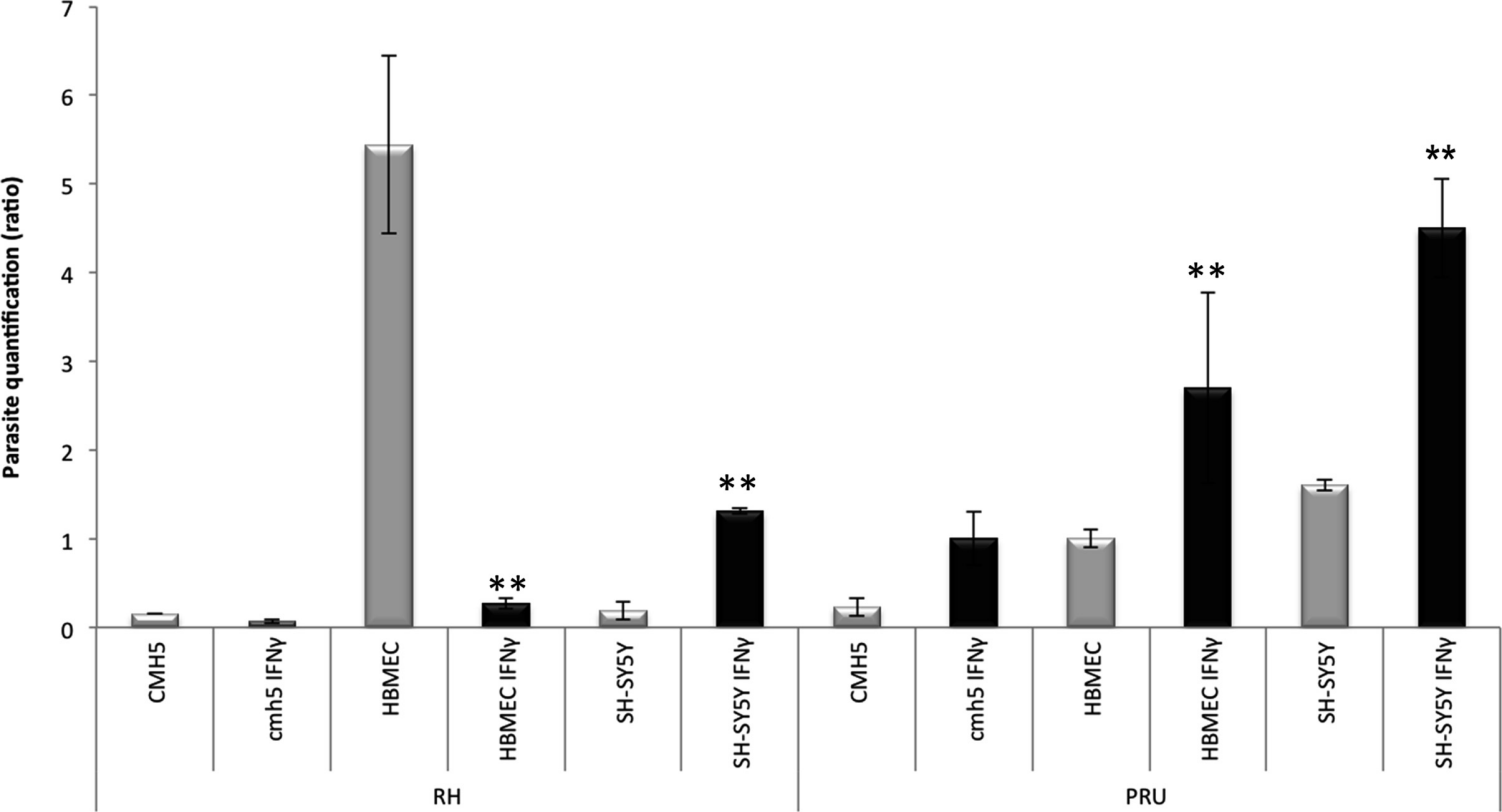
Effect of IFN-γ on parasite multiplication. Microglial cells, endothelial cells and neurons were stimulated by IFN-γ before being infected with the RH or PRU strain of *T. gondii* for 24 h. Parasite burden was quantified by semi-quantitative RT-PCR in stimulated and infected cells (black) and in non-stimulated and infected cells (grey). Parasite quantification was evaluated as follows: final number of tachyzoites/initial number of tachyzoites. Comparison was performed using the Scheirer-Ray-Hare test. **p < 0.01.

At 24 h post-infection, the parasite burden of the RH strain was significantly decreased in IFN-γ-stimulated endothelial cells (*p* < 0.01), but it increased in IFN-γ-stimulated neurons (*p* < 0.01). In contrast, the PRU burden rate remained high in each type of IFN-γ-stimulated cell (*p* < 0.01) (Fig. 1).

## Discussion

Results obtained in this study showed that stimulation by IFN-γ had a significant effect on expression of these immune mediators, decreasing their production compared to non-stimulated infected cells. This decrease was more marked in PRU-infected cells.

More specifically, in PRU-infected microglial cells, IFN-γ caused a decrease of MCP-1, growth factors (G-CSF and GM-CSF), Serpin E1 and sICAM, whereas in microglial cells infected by RH strain, IFN-γ caused a significant decrease of only G-CSF. The chemokine MCP-1 is known for initiating monocyte/macrophage activation which can promote pro-inflammatory immune response against *T. gondii* and it can also participate in the control of *Toxoplasma* infection, as described in human MRC5 fibroblasts [3] and human astrocytes [2]. The decrease of this production under IFN-γ treatment in PRU-infected cells suggests that this cytokine may control a pro-inflammatory immune reaction in the course of a type II infection. This process was confirmed by a decrease of G-CSF and GM-CSF. These two growth factors are involved in dendritic cell activation, granulocyte survival and enhancement of macrophage/microglial function so that the decrease of their production might limit immune cell activation. In addition, these two growth factors were reported to inhibit neutrophil apoptosis during *Toxoplasma* infection [6]. The decrease of growth factor production during PRU infection could promote neutrophil apoptosis and thus reduce the pro-inflammatory reaction induced by this strain. Serpin E1, that also decreased after IFN-γ stimulation of PRU-infected microglial cells, was reported to inhibit the uPA/uPAR pathway which promotes the secretion of MMP-9 active forms by *Toxoplasma-*infected macrophages [23]. After stimulation by IFN-γ, the decrease of Serpin E1 production might promote parasite invasion in these cells.

In PRU-infected endothelial cells, we noted a decrease of IL-6, and of chemokines MCP-1, MIF and GROα, of G-CSF, of Serpin E1, and sICAM. Interleukin-6 contributes to the intracellular control of type I and II parasite strains in mouse neurons [17]. The decrease of IL-6 might be involved in loss of PRU multiplication in these cells. In addition, the decrease of chemokine production could control local inflammation and promote parasite multiplication. MIF is considered as an important protein. It has been described that MIF had a role in controlling *Toxoplasma* parasite burdens via a strong immune inflammation response responsible for intestinal tissue damages. MIF-deficient mice were susceptible to *T. gondii* infection and presented a high parasite burden [13]. In our case, the high parasite burden noted in PRU-infected endothelial cells is facilitated via Serpin E1 and sICAM reduction. In RH-infected endothelial cells, IFN-γ caused a decrease of mostly the same proteins (IL-6, G-CSF and GM-CSF) except MCP-1, MIF, GROα, Serpin E1 and sICAM. These data indicate that the RH strain could cause less local pro-inflammatory reaction than the PRU strain and the normal expression of Serpin E1 and sICAM suggests a role of these proteins in the control of RH strain multiplication in endothelial cells.

In PRU-infected neurons, IFN-γ decreased only growth factors (G-CSF and GM-CSF), but increased sICAM and Serpin E1 production. A different situation was observed when neurons were infected with the RH strain: IFN-γ also increased sICAM and Serpin E1 production, but, contrary to PRU, it increased G-CSF. Moreover, a decrease in MCP-1 levels was observed in RH-infected neurons. The decrease of growth factor production during PRU infection could control pro-inflammatory reactions and promote neutrophil apoptosis of neurons infected by PRU. The high expression of Serpin E1 promotes parasite invasion and multiplication, which could be explained by the high parasite burden in PRU- and RH-infected neurons. For sICAM, this protein was known to have an inhibitory effect on *T. gondii* transmigration across BeWo (human placenta), Caco-2 (human intestine) and MDCK (canine kidney) cells, which can control parasite infiltration [1]. This result might indicate that sICAM could be involved in maintaining infection chronicity and *Toxoplasma* cyst formation [11].

In neurons stimulated by IFN-γ, both RH and PRU multiplication rates remained high at 24 h post-infection, suggesting that this cytokine alone is not sufficient for the control of parasite multiplication in neurons. The effector molecules inducing anti-toxoplasmic activity in neurons have not been specifically identified. In this study, sICAM and Serpin E1 expression indicated that they could be implicated in the increase of intra-neuronal parasite burden. Importantly, in the murine model, astrocytes help neurons to target the parasite, both cell types also serve as immunoregulators via TGF-β signalling pathways [5]. So, we can suggest that neurons alone cannot control tachyzoite multiplication. In contrast, the RH multiplication rate was decreased in human endothelial cells stimulated by IFN-γ, suggesting the implication of IFN-γ in the control of RH multiplication in these human cells. *In vivo*, this effect could be promoted via IFN-γ-activated lymphocytes which had a major role in parasite proliferation control. These cells can activate Th1 immune response and NK cells to limit parasite proliferation in the murine model [10].

In human microglial cells, RH-parasite burden was controlled in unstimulated and stimulated cells. It is known that IFN-γ activates microglial cells to produce NO, which can reduce and control RH intracellular multiplication in the murine model [22]. So, human microglial cells may have the capacity to control *T. gondii* type I strain; the addition of IFN-γ to these infected cells provokes a decrease only in growth factors, which can explain that this cytokine could control the local inflammatory reaction in these infected cells. Unexpectedly, this control of multiplication by IFN-γ in human microglial and endothelial cells was not observed for the type II strain. The PRU burden rate remained high in each type of IFN-γ-stimulated cell, suggesting that the control of PRU proliferation could need other immune mediators such as TNFα, which interact with IFN-γ, as described in murine microglia [14]. Däubener et al. reported that interaction of IFN-γ and TNFα in human brain microvascular endothelial cells induces a strong induction of indoleamine 2,3-dioxygenase (IDO) [9]. Indoleamine 2,3-dioxygenase induction in *Toxoplasma*-infected human brain cells enhances tryptophane degradation and IFN-γ secretion which promote an anti-parasitic effect [8].

In conclusion, IFN-γ is implicated in the decrease of pro-inflammatory reactions in type II-infected microglial and endothelial cells, which promotes parasite persistance. In contrast, IFN-γ may have a role in controlling type I strain multiplication in human microglial and endothelial cells but not in human neurons. Contrary to what has been demonstrated in *in vivo* murine studies, our results show that the PRU strain proliferates in all stimulated and non-stimulated cells, as the result of a specific effect of this strain on host cell responses or of absence of the immune protein and cellular interaction that takes place *in vivo.* Further studies integrating these interactions would be needed to confirm these results.

## Conflict of interest

None.
